# Targeting Mll1 H3K4 methyltransferase activity to guide cardiac lineage specific reprogramming of fibroblasts

**DOI:** 10.1038/celldisc.2016.36

**Published:** 2016-10-11

**Authors:** Liu Liu, Ienglam Lei, Hacer Karatas, Yangbing Li, Li Wang, Leonid Gnatovskiy, Yali Dou, Shaomeng Wang, Li Qian, Zhong Wang

**Affiliations:** 1 Department of Cardiac Surgery, Frankel Cardiovascular Center, The University of Michigan, Ann Arbor, MI, USA; 2 Faculty of Health Sciences, University of Macau, Macau SAR, China; 3 Department of Internal Medicine, University of Michigan School of Medicine, Ann Arbor, MI, USA; 4 Department of Pharmacology, University of Michigan School of Medicine, Ann Arbor, MI, USA; 5 Department of Medicinal Chemistry, University of Michigan College of Pharmacy, Ann Arbor, MI, USA; 6 Department of Pathology and Laboratory Medicine, University of North Carolina, Chapel Hill, NC, USA; 7 McAllister Heart Institute University of North Carolina, Chapel Hill, NC, USA; 8 Lineberger Comprehensive Cancer Center, University of North Carolina, Chapel Hill, NC, USA; 9 Department of Pathology, University of Michigan Medical School, 1301 Catherine, Ann Arbor, MI, USA

**Keywords:** adipocyte, cardiomyocyte, Cardiac reprogramming, H3K4 methyltransferase, Mll1 inhibitor

## Abstract

Generation of induced cardiomyocytes (iCMs) directly from fibroblasts offers a great opportunity for cardiac disease modeling and cardiac regeneration. A major challenge of iCM generation is the low conversion rate. To address this issue, we attempted to identify small molecules that could potentiate the reprogramming ability towards cardiac fate by removing inhibitory roadblocks. Using mouse embryonic fibroblasts as the starting cell source, we first screened 47 cardiac development related epigenetic and transcription factors, and identified an unexpected role of H3K4 methyltransferase Mll1 and related factor Men1 in inhibiting iCM reprogramming. We then applied small molecules (MM408 and MI503) of Mll1 pathway inhibitors and observed an improved efficiency in converting embryonic fibroblasts and cardiac fibroblasts into functional cardiomyocyte-like cells. We further observed that these inhibitors directly suppressed the expression of Mll1 target gene *Ebf1* involved in adipocyte differentiation. Consequently, Mll1 inhibition significantly decreased the formation of adipocytes during iCM induction. Therefore, Mll1 inhibitors likely increased iCM efficiency by suppressing alternative lineage gene expression. Our studies show that targeting Mll1 dependent H3K4 methyltransferase activity provides specificity in the process of cardiac reprogramming. These findings shed new light on the molecular mechanisms underlying cardiac conversion of fibroblasts and provide novel targets and small molecules to improve iCM reprogramming for clinical applications.

## Introduction

Heart disease is the leading cause of death in the United States and around the world [1]. On heart injuries, such as myocardial infarction, millions of cardiomyocytes undergo irreversible necrosis and infarct areas are replaced with fibroblasts and further differentiate into nonfunctional tissues like fibrotic scar [2] or adipose tissue [3, 4]. Recently, targeting fibroblasts by introduction of three transcription factors Gata4, Mef2C and Tbx5 (G, M and T), and reprogramming them into cardiomyocyte-like cells have shown therapeutic potential [5–8]. However, direct reprogramming of fibroblasts into functional cardiomyocytes with high efficiency remains a challenge.

Numerous strategies have been applied to achieve better efficiency of induced cardiomyocyte (iCM) reprogramming. One strategy is to supply/introduce additional transcription factors to the cocktail. It has been shown that adding Hand2 [9], Nkx2-5 [10], Mesp1, MyoCD or Baf60c [11] can increase the reprogramming efficiency and/or improve functional properties of iCMs. Although multiple combinations have been used for reprogramming, Mef2C, Gata4 and Tbx5 (MGT) seem to be irreplaceable. Furthermore, stoichiometric expression of G, M, T proteins also affects the efficiency of MGT-mediated reprogramming [7]. Other strategies include application of Akt1 [12] or TGF-beta and Rho-associated kinase [13] to inhibit pro-fibrotic signaling. Two microRNAs, mir-1 and mir-133, when used in combination with transcription factors, could also increase iCM reprogramming efficiency [13–15].

Cardiac reprogramming is a process involving extensive epigenetic changes [16]. Therefore, modulating epigenetic changes is an effective strategy to improve efficiency. Successful examples include the application of inhibitors of Ezh2 methyltransferase and G9a histone methyltransferase [17] and shRNA-mediated knockdown of Bmi1, a regulatory component of polycomb complex involved in gene silencing [18].

Another potential novel strategy to achieve more specificity is to block conversion of fibroblasts into non-myocytes during iCMs induction. The induction of unexpected cell types in reprogramming appears to be rather common. It has been observed that Ascl1 promotes both neuron and myocyte identities in direct reprogramming from fibroblasts to neurons [19]. A recent study also shows that Yamanaka factor-directed iPSC reprogramming induces extraembryonic endoderm stem cells [20]. During iCM reprogramming, the binding of transcription factor to non-cardiac specific promoter can induce non-cardiac gene expression and likely leads to differentiation of unexpected cell types [21, 22]. Therefore, identifying epigenetic factors and related small molecules to block lineage conversion into non-cardiomycoyte cell types may in turn promote iCMs formation.

In this report, we used a gain-of-function screen to identify regulatory factors involved in iCM reprogramming, which revealed an unexpected role of Mll1 H3K4 methyltransferase and related co-activator Men1 in inhibiting iCM formation. We demonstrated that addition of Mll1 inhibitor MM408 converted embryonic fibroblasts into functional cardiomyocyte-like cells with a much higher efficiency. Furthermore, we identified Ebf1, a key factor involved in adipocyte differentiation, as a target of Mll1 during iCM reprogramming. Consequently, we observed that MM408 treatment during reprogramming inhibited adipocyte related gene expression and adipocyte formation and guided more cells into cardiomyocyte lineage. These findings provide novel insights into the molecular mechanisms of iCM reprogramming and identify small molecules for improved iCM generation.

## Results

### Gain-of-function screen identified menin1 (Men1) as a barrier to iCM reprogramming

**Figure 1:**
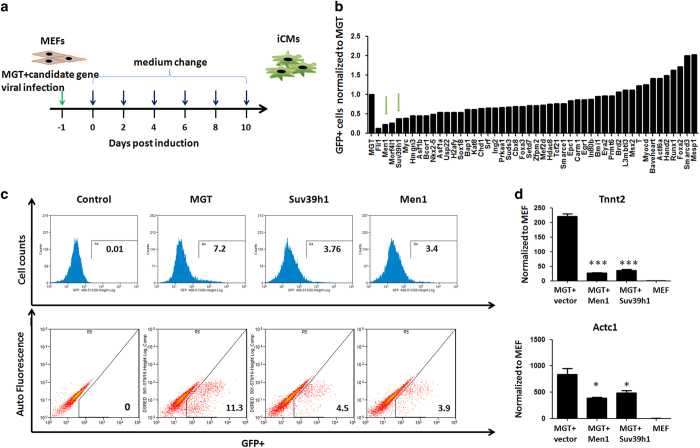
Gain-of-function screen identified Men1 as a barrier of iCM reprogramming. (**a**) A schematic diagram of the gain-of-function screen strategy. Cloned individual gene was transduced into MEFs derived from a transgenic αMHC-GFP reporter mouse together with retroviruses expressing polycistronic MGT. Medium was changed every 2 days. Activation of GFP was evaluated 10 days after transduction by flow cytometry. (**b**) Column graph of normalized percentages of αMHC-GFP+, green arrow pointing at the fold change of Men1 and Suv39h1. (**c**) Representative flow cytometry results of Men1 and Suv39h1 groups shown by histogram (upper panel) and flow plots (lower panel). For the MGT plus empty pCW57.1 group, 11% GFP positive cells were detected by flow cytometry. For MGT+ Men1 and MGT+ Suv39h1 groups, 3.9 and 4.5% of αMHC-GFP+ cells were detected, respectively. (**d**) Relative expression of cardiomyocytes marker genes Tnnt2 and Actc1 in MEFs infected with MGT combined with Men1, Suv39h1 or empty vector after 10 days. Untransduced MEFs were used as a negative control (MEF). Error bars indicate mean±s.e.m.; *P<0.05, **P<0.01, ***P<0.001.

To identify potential transcription and epigenetic regulators of iCM reprogramming, we employed a gain-of-function approach to explore the function of 47 genes (Figure 1a). On the basis of RNA-seq data of cardiac cell differentiation [23], we cloned 47 transcription and epigenetic regulators highly expressed in the cardiac progenitor and neonatal cardiomyocytes stage into a pCW57.1 based lentivirus vector pool (Supplementary Table S1). The individual genes were then transduced into mouse embryonic fibroblasts (MEFs) from a transgenic α-muscle heavy chain (αMHC)-green fluorescent protein (GFP) reporter mouse [5, 7] together with retroviruses expressing polycistronic Mef2c/Gata4/Tbx5 (MGT) [7]. Ten days after transduction, activation of GFP was used to evaluate the efficiency of reprogramming (Figure 1b). For the MGT plus empty pCW57.1 group, we observed 11% GFP positive cells as reported previously, indicating similar successful rate for reprogramming (Figure 1c). Furthermore, we also observed the enhancing effect of Hand2, Mesp1 and Baf60c as reported [9, 11] (Figure 1b). Among the 47 candidate regulators, overexpression of epigenetic regulators Men1 and Suv39h1 resulted in fivefold and threefold reduction in αMHC-GFP+ cells (Figure 1b). Flow cytometry detected 3.9 and 4.5% of αMHC-GFP+ cells in MGT+ Men1 and MGT+ Suv39h1 groups, respectively (Figure 1c). Quantitative real-time PCR (QPCR) results indicated that key cardiac genes Actc1 and Tnnt2 were reduced following Men1 or Suv39h1 overexpression (Figure 1d). These results showed that Men1 and Suv39h1 were barriers to iCM reprogramming.

### Men1 inhibited reprogramming through Mll1

**Figure 2:**
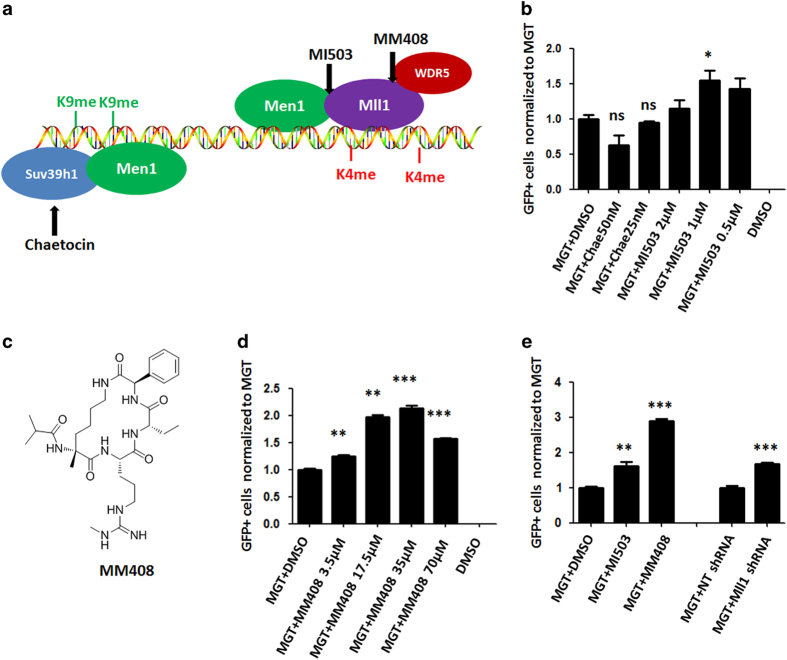
Mll1 inhibitors significantly enhanced cardiac reprogramming. (**a**) A diagram depicting the interaction of Men1 with Mll1 complex and Men1 with Suv39h1. The target sites of small molecules have been marked with arrows. (**b**) The effect of Suv39h1 inhibitor chaetocin and Men1 inhibitor MI503 treatment on iCM reprogramming. Relative fold changes of αMHC-GFP+ were normalized to MGT induction with DMSO treatment. (**c**) The chemical structure of MM408. (**d**) Optimization of Mll1 inhibitor MM408 treatment for improving reprogramming indicated by the fold change of αMHC-GFP+ cells. (**e**) The effect of MI503, MM408 and Mll1 shRNA on iCM reprogramming indicated by the fold change of αMHC-GFP+ cells in MGT-transduced MEFs. Non-targeting (NT) shRNA was used as control for Mll1 shRNA. Error bars indicate mean±s.e.m.; *P<0.05, **P<0.01, ***P<0.001.

Men1 is related to histone marks H3K9me3 and H3K4me3 [24, 25], and Suv39h1 is a regulator for H3K9me3 [26] (Figure 2a). As H3K9me3 has been known as a repressive marker, we postulated that Men1 might inhibit reprogramming through activating H3K9 methyltransferase activity. Therefore, we applied Suv39h1 inhibitor chaetocin [27] and Men1 inhibitor MI503 [28] in our reprogramming assays. Surprisingly, we did not observe any improvement in reprogramming with addition of Suv39h1 inhibitor chaetocin, but observed 1.5-fold increase with Men1 inhibitor MI503 (Figure 2b). Because MI503 targets Men1 by blocking the binding of Men1 to the Mll1 and Mll2 complex (Figure 2a) [28], Men1 likely affected reprogramming by activating Mll1/2-related H3K4 methyltransferase activity, instead of repressing H3K9me methyltransferase activity.

Histone H3K4 methylations are generally associated with gene activation, therefore our finding that inhibiting the activity of Mll1 H3K4 methyltransferase enhanced cardiac reprogramming was surprising. To confirm that MI503 increased reprogramming through inhibiting Mll1-related H3K4me3 activity, we applied a novel Mll1 complex-specific inhibitor in our system. MM408 [29] compound is a modified version of the previously reported MM401 [30] that disrupts the interaction of WDR5 with Mll1 with higher binding affinity (Figure 2c). It specifically inhibits Mll1-related H3K4 methyltransferase activity without affecting the activities of other Mll family proteins. Indeed, MM408 inhibitor increased reprogramming efficiency in a dose dependent manner (Figure 2d). Overall, 17.5 μm or 35 μm MM408 resulted in the highest iCM conversion rate, whereas 70 μm MM408 resulted in potential toxicity and lowered the conversion rate. Also, 17.5 μm MM408 led to a significantly higher iCM conversion rate compared with optimized 1 μm MI503 (Figure 2e). In addition, Mll1 shRNA knockdown also resulted in elevated iCM conversion rate (Figure 2e). Collectively, these results suggested that inhibiting Mll1 activities by applying small molecules MI503 and MM408 increased the reprogramming efficiency.

### Mll1 inhibitors significantly enhanced cardiac reprogramming

**Figure 3:**
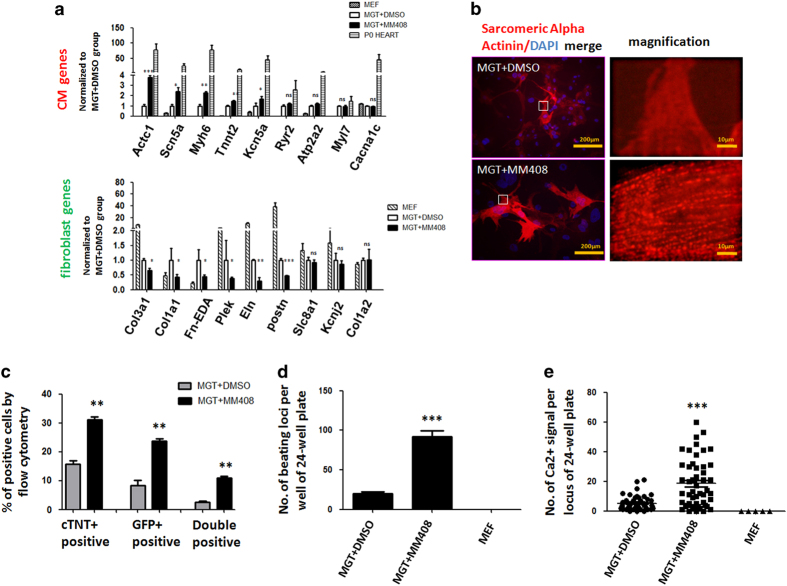
Mll1 inhibitor MM408 improved functional properties of induced cardiomyocytes. (**a**) Relative expression of CM and fibroblast marker genes in neonatal whole heart isolated from postnatal day 0 (P0) mice and DMSO or MM408-treated iCMs 10 days after MGT transduction. Statistic analyses were performed between MM408- treatment and DMSO-treatment groups. (**b**) Immunocytochemistry (ICC) of cardiac markers α-actinin of MGT-transduced cells with or without MM408 treatment by fluorescence microscopy (400×). The right panels were enlarged areas from the left panels. (**c**) Quantification for αMHC-GFP+ and cTnT+ cells or double positive cell detected by flow cytometry 10 days after transduction. (**d**) Quantification of the number of beating iCMs loci with indicated viral infection and small molecule treatment for 4 weeks (*n=*10). (**e**) Quantification of spontaneous Ca^2+^ oscillations cells per field with indicated viral infection and small molecule treatment for 4 weeks (*n=*50 from 10 wells). Untransduced MEFs were used as a negative control (MEF). Error bars indicate mean±s.e.m.; *P<0.05, **P<0.01, ***P<0.001.

We next investigated the effect of Mll1 inhibition on the functional properties of iCMs. As MM408 resulted in the highest iCM conversion rate compared with other inhibitors, we focused on MM408 in our following experiments. Owing to its near highest iCM conversion rate and low cell toxicity, 17.5 μm MM408 was used in these experiments (Figure 2). We first performed molecular characterizations of the MM408-treated and untreated cells by examining the expression of a panel of sarcomeric, contractility and ion channel genes. The results revealed a higher expression of these functionally important cardiac genes especially Actc1, Scn5a and Myh6 in the MM408-treated group (Figure 3a). In addition, MM408-treated cells showed reduced expression of genes associated with the fibroblast lineage. To test whether treatment of MM408 promotes iCM maturation, we then examined the structural properties using immunostaining against cardiac Troponin T (cTnT) and sarcomeric α-actinin, two specific components of sarcomere in cardiomyocyte. Immunostaining against these two proteins could clearly show the sarcomeric structure. Treatment with MM408 for 2 weeks significantly increased the number of cells with assembled sarcomeric structures compared with the control group (Figure 3b; Supplementary Figure S1). Flow cytometric analyses further confirmed that MM408 induced higher cTnT expression. In particular, we observed that 25% of the cells became GFP positive and 30% of the cells became cTnT positive in MM408-treated group, more than twofold increase over non-MM408-treated group (Figure 3c). Notably, 10% GFP and cTnT double positive cells were observed, indicating a more than threefold increase over controls.

Furthermore, we evaluated the presence of beating iCMs in our reprogramming assays using the beating protocol as reported [31]. Spontaneously contracting cells were apparent 3 weeks after MM408 treatment indicating that MM408 treatment significantly increased beating iCMs (Supplementary Movies S1 and S2). Approximately 92 beating loci per well were identified in MM408-treated group 4 weeks after transduction (Figure 3d). In contrast, only 20 beating loci were observed in non-MM408-treated group. To further assess the functionality of MM408-treated iCMs, we next analyzed intracellular Ca^2+^ flux by Rhod3 dye labeling 4 weeks after reprogramming. MM408-treated iCMs showed significantly higher spontaneous Ca^2+^ oscillations with various frequencies compared with control group (Figure 3e; Supplementary Movies S3 and S4), as previously reported in our reprogrammed iCMs [7, 18]. By using neonatal cardiac fibroblast instead of MEFs transduced with MGT, we also observed more beating loci in MM408-treated group compared with non-MM408-treated group (Supplementary Figure S2; Supplementary Movies S5 and S6). These results revealed that MM408 not only increased cardiac gene expression, but also enhanced iCM function.

### Mll1 inhibitor guided cardiac reprogramming by suppressing adipocyte formation

**Figure 4:**
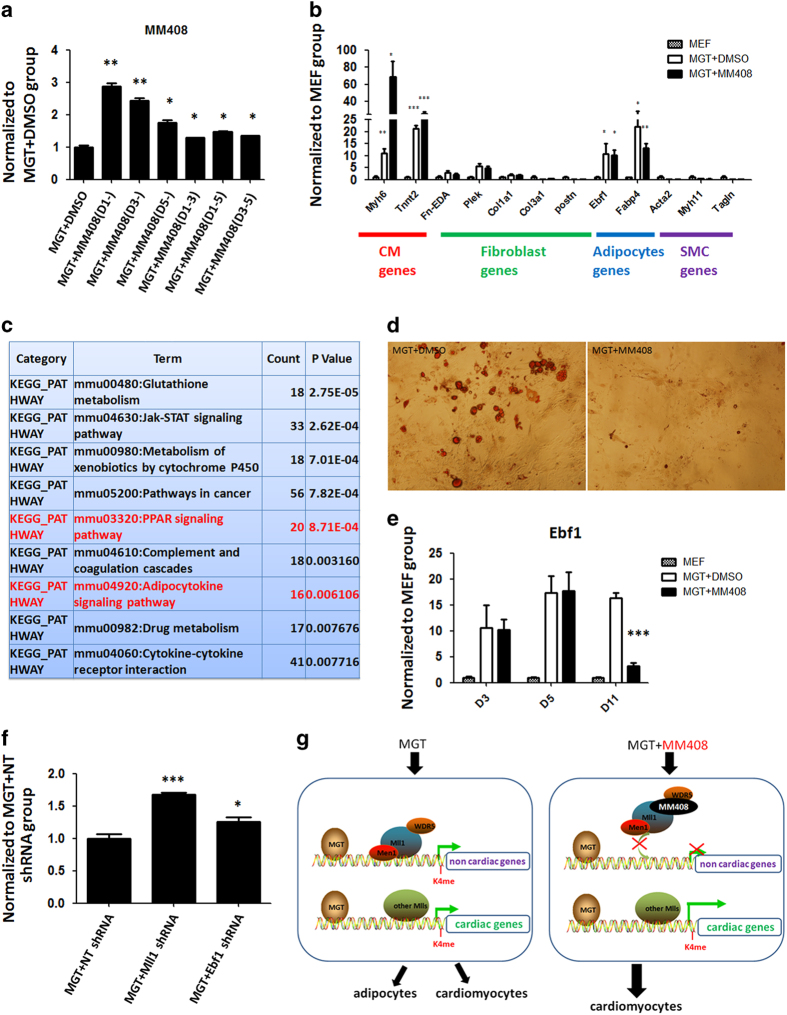
Mll1 inhibitor guided cardiac reprogramming by suppressing ectopic adipocyte related gene expression. (**a**) The effect of timing and duration of WDR5 inhibitor MM408 treatment on iCM reprogramming, as shown by the fold change of αMHC-GFP+ cells after MGT transduction. Mll1 inhibitor was added with various time durations after viral transduction: D1-, from day 1 until detection; D3-, from day 3 until detection; D5-, from day 5 until detection; D1-3, from day 1 to day 3; D1-5, from day 1 to day 5; D3-5, from day 3 to day 5. (**b**) Relative expression of cardiomyocyte (CM), fibroblast, adipocyte and smooth muscle cells (SMC) marker genes in early stage of iCM reprogramming with either MM408 or DMSO treatment 3 days after MGT transduction. Statistic analyses were performed between MEF with MM408-treated or DMSO-treated groups. Untransduced MEFs were used as a negative control (MEF). (**c**) Gene Ontology (GO) enrichment analysis of down-regulated genes in Mll1^−/−^ MEFs. The list shows enriched pathway ordered by *P*-value. The pathways related with Pparg and adipocyte lineage were marked in red. (**d**) Oil Red O stain of adipocyte cells in MGT-transduced MEFs 14 days after transduction with or without MM408 treatment. (**e**) Expression of Ebf1 during reprogramming in MGT-transduced MEFs, with or without MM408 treatment. The *P*-value reflecting the statistical significant difference of the expression of Ebf1 at D11 was 0.0004. Untransduced MEFs were used as a negative control (MEF). (**f**) The effect of Mll1 or Ebf1 knockdown on improving iCM efficiency indicated by the fold change of αMHC-GFP+ cells. Non-targeting (NT) shRNA was used as a control for Mll1 or Ebf1 shRNA. (**g**) A working model of MM408 mediated cardiac reprogramming. Mll1 inhibitor MM408 promoted iCM conversion by suppressing adipocyte lineage gene expression. Error bars indicate mean±s.e.m.; *P<0.05, **P<0.01, ***P<0.001.

To characterize the cellular and molecular mechanisms of MM408/Mll1 action during iCM induction, we set out to determine the timing of Mll1 inhibitor-mediated effects on iCM reprogramming (Figure 4a). Fibroblasts were transduced with MGT on day 0, and MM408 compound was added to the culture medium at various time points. The most effective time-window was to add MM408 from day 1, resulting in three times as many GFP+ cells as negative control. These results indicated that MM408 exerted its effect from the initiation of reprogramming, consistent with the idea that epigenetic repatterning occurs at early stage of reprogramming [16, 18]. Therefore, we attempted to identify the direct target(s) of Mll1 in fibroblasts that mediate(s) its effect on iCM reprogramming.

Given the fact that M, G and T are all potent transcription factors that regulate multiple biological processes during early embryonic development, it is likely that during MGT-mediated iCM formation, reprogramming factors not only activate cardiac specific gene expression but also induce non-cardiac lineage gene expression. To test this hypothesis, we examined the expression of fibroblast-, adipocyte- and SMC-related genes at the early stage of reprogramming (3 days post transduction). We found that in the early stage of reprogramming, overexpression of MGT in fibroblasts led to not only elevated cardiac gene expression but also increased adipocyte gene expression 3 days post transduction. In contrast, fibroblast or SMC-related genes in MM408-treated and DMSO-treated groups did not change (Figure 4b). By comparing the expression profiles of Mll1^−/−^ MEFs versus Mll1^+/+^ MEFs [32] with gene ontology analysis, we discovered that the down-regulated genes were highly related to adipogenesis and peroxisome proliferator-activated receptor pathways (Figure 4c). Consistent with this observation, in Mll1^−/−^ MEFs, numerous genes related to those functions were down-regulated. More importantly, we observed adipocyte-like cells and increased adipocyte formation detected by Oil Red O stain 14 days after MGT transduction (Figure 4d). These results suggested that during reprogramming, ectopic expression of adipocyte lineage genes might interfere with the acquisition of cardiomyocyte cell fate in fibroblasts.

Therefore, we set out to test whether Mll1 inhibitors enhanced cardiac reprogramming by inhibiting adipocyte formation. On the basis of the fold change of expression and our previous report that Mll1 directly binds to Ebf1 gene locus in MEFs and embryonic stem cells [32, 33], we propose that adipogenesis-related Ebf1 could be a key target of Mll1 inhibitor in iCM reprogramming as Ebf1 expression was elevated during reprogramming (Figure 4e). In contrast, when MM408 was applied during iCM induction, expression of Ebf1 was dramatically decreased at day 11 post transduction (Figure 4e). Furthermore, adipogenesis marker genes Fabp4 and Pparg also exhibited a decreased expression after MM408 treatment (Supplementary Figure S3). Consistent with the decrease of adipogenesis-related gene expression, Oil Red O staining detected a significant decrease in adipocyte formation with MM408 treatment (Figure 4d). To directly test if blocking adipocyte lineage gene expression could promote iCM formation, we performed RNAi-mediated knockdown of Ebf1 and Mll1 during iCM formation. Indeed, knockdown of Ebf1 or Mll1 increased iCM reprogramming efficiency (Figure 4f), indicating that Mll1 inhibitor repressed adipocyte formation mainly through blocking the expression of Ebf1. Altogether, these results strongly suggested that inhibition of Mll1 promotes iCM conversion by suppressing adipocyte lineage gene expression (Figure 4g).

## Discussion

We report here an unexpected role of Mll1 and related factor Men1 in cardiac reprogramming. Application of novel small molecules MM408 and MI503, inhibitors of Mll1 and Men1, resulted in much higher efficiency of iCM formation from embryonic fibroblasts. Mechanistically, these inhibitors directly suppressed the expression of Mll1 target gene Ebf1, a key gene involved in adipocyte differentiation. In parallel with Mll1 inhibition, we observed decreased formation of adipocytes in our iCM assays. Therefore, Mll1 inhibitors increased iCM efficiency likely through suppressing the expression of adipocyte lineage genes. Our studies show that targeting Mll1-related H3K4 methyltransferase activity with Mll1 inhibitors provides more specificity in the process of iCM induction.

Given the well-established antagonistic roles of TrxG and PcG complexes in development [34], our discoveries that inhibiting the activities of both PcG component Bmi1 and TrxG component Mll1 enhance iCM formation are surprising. However, our further studies indicate that Mll1 and Bmi1 exert their respective activating and repressive functions via distinct mechanisms [18]. Specifically, reduced Bmi1 expression leads to increased levels of the active histone mark H3K4me3 and reduced levels of repressive H2AK199ub at cardiogenic loci, and de-repression of cardiogenic gene expression [18]. In contrast, as reported here, inhibiting Mll1 activity leads to decreased expression of genes involved in adipocyte formation and consequent low induction of adipocytes. Therefore, seemingly opposite functions of epigenetic factors may work together to facilitate reprogramming or transdifferentiation. Future studies will determine whether combined inhibition of Mll1 and Bmi1 could synergistically increase iCM conversion in a time-window dependent fashion.

To our knowledge, this is the first study to show that epigenetic factor/inhibitor can enhance cardiac reprogramming by blocking conversion of fibroblasts into non-myocyte cell types during iCMs induction. MM408, an inhibitor of Mll1-WDR5 interaction, is able to increase iCM formation by inhibiting adipogenesis. Our studies reported here point to a new direction in enhancing iCM conversion. Instead of boosting iCM conversion directly by increasing the cardiac gene expression, actively incorporating strategies to inhibit conversion of fibroblasts into other related cell types may also increase iCM formation.

Mechanistically, this MM408 mediated Mll1 inhibition is achieved, at least partially, by blocking the expression of Mll1 target Ebf1, a key gene involved in adipocytes differentiation [35]. Our discovery is consistent with a number of Mll1 studies in cell lineage development, which show that Men1 directly targets Pparg [36] through Mll to facility adipogenesis. Our results also show that in addition to Pparg, Ebf1 is also involved in adipocyte differentiation as a direct Mll1 target. Future research focusing on the epigenetic changes during the whole reprogramming process will reveal the mechanistic details of how Mll1 regulates cardiac reprogramming.

Our study shows that MGT not only induces iCM but also increases adipocyte formation. The molecular mechanism of MGT-mediated adipogenesis will be explored in the future. One potential reason might be that iCM and adipocytes are developmentally related. It has been shown that Myf5 positive myogenic precursors can differentiate into both adipocytes and muscle cells. Mature adipocytes [37] and adipocyte derived stem cells [38] possess the ability to differentiate into functional cardiomyocytes. These studies suggest close functional and original connections between adipocyte and muscle cell. Indeed, the observation that induction of unexpected cell types in reprogramming appears to be a common theme, as has been reported recently in neuron [19] and iPSC [20] reprogramming.

We expect that applications of chemical approaches for reprogramming and transdifferentiation represent a highly desirable strategy in heart regeneration to treat heart attack and associated heart failure. In fact, a couple of recent studies show the feasibility that cardiomyocyte-like cells can be obtained from embryonic fibroblasts with only small molecule treatment *in vitro* [39, 40]. Future studies combining MM408 described here with small molecules and candidate molecules identified from other studies should convert fibroblasts into functional and more mature beating cardiomyocytes with high efficiency.

## Materials and Methods

### Mouse lines

The transgenic mice harboring GFP under control of α-MHC promoter was used to derive MEFs as previous reported [7]. Another transgenic mice harboring mCherry under control of modified α-MHC promoter was purchased from Jackson’s lab (021577).

All animal-related procedures were approved by the Institutional Animal Care and Use Committee of the University of Michigan and are consistent with the National Institutes of Health Guide for Use and Care of Animals.

### Plasmids

The retroviral polycistronic constructs vectors encoding mouse Mef2c, Gata4 and Tbx5 in pMXs based vectors were constructed as described previously [7]. DNA fragment containing Mef2c, Gata4 and Tbx5 sequentially were separated by oligonucleotides encoding P2A and T2A peptides constructed into a polycistronic constructs vector. The shRNA lentivirus vector for Ebf1 (pGipZ, V2LMM-20601), Mll1 (pGipZ, V2LMM-96428) were from Vector Core of University of Michigan.

### Primary cell isolation

MEFs (isolated at E13.5) were prepared as previously described. Briefly, embryos were harvested from transgenic mice of α-MHC-GFP or α-MHC-mCherry at 13.5 days post coitum followed by decapitation and removal of internal organs, including the heart. The tissue was minced and digested with TrypLE Express Enzyme (Thermo Fisher Scientific, Waltham, MA, USA). Cells were resuspended in MEFs medium (10% FBS and 2 mM l-glutamine contained DMEM medium) and plated onto one 10 cm dish per embryo. Cells were regularly passaged at 1:3 (passage 1). Passage 3 MEFs were used for reprogramming.

### Inhibitor information

Men1 inhibitor was from Active Biochem (MI503, A-1399), Suv39h1 inhibitor (chaetocin, sc-200893) was from Santa Cruz, Mll1/WDR5 inhibitor (MM408) was from Dr. Shaomeng Wang’s lab.

### Retrovirus and lentivirus preparation

Retrovirus vectors were packaged into Plat-E cells using Lipofectamine 2000 (Invitrogen, Carlsbad, CA, USA) to deliver 10 μg in 1.5 ml Opti-MEM (Invitrogen) to 80% confluent 10 cm plates of Plat-E cells with 6 ml of Opti-MEM. 4-6 h later, Opti-MEM was changed into 10ml fresh MEFs medium. Forty-eight hours and seventy-two hours after transfection, viral medium was harvested twice and filtered through a 0.45-mm cellulose filter. The harvested virus containing medium was added 1/5 vol of 40% PEG8000 solution to make a final concentration of 8% PEG8000. The mixture was kept at 4 °C overnight and spined at 3000 *g*, 4 °C, 30 min to get concentrated white pellet. Fresh MEFs medium was added to resuspend virus. The viral supernatant was mixed with polybrene (Sigma, St Louis, MO, USA) to a final concentration of 8 μg ml^−1^.

Lentiviral vectors were packaged into HEK293T cells (ATCC) using Lipofectamine 2000 (Invitrogen) to deliver 10 μg of the lentiviral backbone plasmid, 6 μg psPAX2, and 4 μg pMD2.G in 1.5 ml Opti-MEM (Invitrogen) to 80% confluent 10 cm plates of 293 cells with 6 ml of Opti-MEM. 4-6 h later, Opti-MEM was changed into 10 ml fresh MEFs medium. The virus was harvested as descried before.

### Direct conversion of fibroblasts to iCMs

Direct conversion of MEFs was completed using a protocol similar to that previously described [7]. Fresh fibroblasts were plated on tissue culture dishes at a density of 10 000 cells cm^−^^2^ before transduction. Fibroblasts were infected with freshly made viral mixture containing polybrene (Sigma) 24 h post plating. Twenty-four hours later, the viral medium was replaced with induction medium composed of DMEM/199 (4:1) (Gibco, Waltham, MA, USA), 10% FBS (Gemini, West Sacramento, CA, USA) and antibiotics (Gibco). Medium was changed every 2–3 days until cells were examined.

### Immunocytochemistry

Samples were fixed using 4% PFA with 0.1% TritonX-100. The following primary antibodies were used: mouse anti-cardiac Troponin T (Thermo Scientific, Waltham, MA, USA), mouse anti sarcomeric a-actinin (Abcam, Waltham, MA, USA). For Oil Red O staining, dishes were washed in PBS three times and cells fixed in 3.7% formaldehyde for 1h, followed by staining with Oil Red 0 for 1h; after staining, plates were washed twice in water and photographed. The final Oil Red O solution used consists of 60% original Oil Red O solution (0.3% Oil Red O in 60% isopropanol (Sigma)) diluted with 40% DI water.

### Flow cytometry

For fluorescence measurements only, data from 10 000 single cell events were collected using a standard MoFlo Astrios flow cytometer (Immunocytometry Systems; Becton Dickinson, Detroit, MI, USA). Data were analyzed using Summit (Becton Dickinson).

### Quantitative real time PCR (QPCR)

Total RNA was extracted using Trizol Reagent (Invitrogen) according to the manufacturer’s instructions. RNA integrity was determined using formaldehyde denaturalization agarose gel electrophoresis. RNA concentrations were measured with the smartspec plus spectrophotometer (BioRad, Hercules, CA, USA). RNA was reverse transcribed using iScript cDNA Synthesis Kit (BioRad). QPCR was performed using StepOne Real-Time PCR System (Thermo Fisher Scientific).

Primer oligonucleotides were synthesized by Invitrogen and are listed in Supplementary Table S2.

### Statistical analysis

Results were presented as mean±s.e.m. Statistical significance between groups was analyzed by one-way ANOVA followed by the Student–Newman–Keuls multiple comparisons tests. A *P*-value of <0.05 was considered significant. Each experiment was performed at least twice.
