# Supplementary material for: Targeting Mll1 H3K4 methyltransferase activity to guide cardiac lineage specific reprogramming of fibroblasts
Source: Cell Discov. 2016 Oct 11;2:16036–. doi: 10.1038/celldisc.2016.36 (PMC5113048; doi:10.1038/celldisc.2016.36)

Supplementary Table 1. Genes used for screening in this study.

| | | | | |
|---|---|---|---|---|
| Fli1 | Usp22 | Cbx8 | In80b | Actl6a |
| Men1 | H2afy | Foxa3 | Bmi1 | Hdac8 |
| Morf4l1 | Sox18 | Setd7 | Eya2 | Hand2 |
| Suv39h1 | Bap1 | Zfpm2 | Prmt6 | Runx1 |
| Myc | Kat8 | Mef2d | Brd2 | Foxa2 |
| Hmgn3 | Chd1 | Tcf21 | L3mbtl3 | Smarcd3 |
| Asf1b | Srf | Smarce1 | Msx2 | Mesp1 |
| Bcor1 | Ing2 | Epc1 | T | |
| Nkx2-5 | Prkaa1 | Carm1 | Myocd | |
| Asf1a | Suds3 | Egr1 | Braveheart | |

Supplementary Table 2. Primers and sequences used for QPCR detection.

| | |
|---|---|
| QPCR-Plek-F | CAAAGCGGATCAGGGAGGG |
| QPCR-Plek-R | TTGACAAGGGCTAGTGAGAGT |
| QPCR-Ebf1-F | GCATCCAACGGAGTGGAAG |
| QPCR-Ebf1-R | GATTTCCGCAGGTTAGAAGGC |
| QPCR-Ryr2-F | ACGGCGACCATCCACAAAG |
| QPCR-Ryr2-R | AAAGTCTGTTGCCAAATCCTTCT |
| QPCR-postn-F | CCTGCCCTTATATGCTCTGCT |
| QPCR-postn-R | AAACATGGTCAATAGGCATCACT |
| QPCR-Fabp4-F | GGAAGACCACTCGCATTCCTT |
| QPCR-Fabp4-R | GTAATCAGCAACCATTGGGTCA |
| QPCR-Pparg-F | TCGCTGATGCACTGCCTATG |
| QPCR-Pparg-R | GAGAGGTCCACAGAGCTGATT |
| QPCR-Fn-EDA-F | TTGATTTCTTTCATTGGTCCTGTCTT |
| QPCR-Fn-EDA-R | AAACAGAAATGACCATTGAAGGTTTG |
| QPCR-Col1a1-F | AGACATGTTCAGCTTTGTGGAC |
| QPCR-Col1a1-R | GCAGCTGACTTCAGGGATG |

| | |
|---|---|
| QPCR-Tagln-F | CAACAAGGGTCCATCCTACGG |
| QPCR-Tagln-R | ATCTGGGCGGCCTACATCA |
| QPCR-Acta2-F | GTCCCAGACATCAGGGAGTAA |
| QPCR-Acta2-R | TCGGATACTTCAGCGTCAGGA |
| QPCR-Myh11-F | GTGTGGTGGTCAACCCCTAC |
| QPCR-Myh11-R | GATGTGAGGCGGCATCTCAT |
| QPCR-Myl7-F | GGCACAACGTGGCTCTTCTAA |
| QPCR-Myl7-R | TGCAGATGATCCCATCCCTGT |
| QPCR-Kcna5-F | TCCGACGGCTGGACTCAATAA |
| QPCR-Kcna5-R | CAGATGGCCTTCTAGGCTGTG |
| QPCR-Cacna1c-F | TCCCGAGCACATCCCTACTC |
| QPCR-Cacna1c-R | ACTGACGGTAGAGATGGTTGC |
| QPCR-Slc8a1-F | CTTCCCTGTTTGTGCTCCTGT |
| QPCR-Slc8a1-R | AGAAGCCCTTTATGTGGCAGTA |
| QPCR-Atp2a2-F | GAGAACGCTCACACAAAGACC |
| QPCR-Atp2a2-R | CAATTCGTTGGAGCCCCAT |
| QPCR-Kcnj2-F | ATGGGCAGTGTGAGAACCAAC |
| QPCR-Kcnj2-R | TGGACTTTACTCTTGCCATTCC |
| QPCR-Col2a1-F | CCTCAAGGCAAAGTTGGTCCT |
| QPCR-Col2a1-R | CTCCCGTCTCACCGTCTTTT |
| QPCR-Col3a1-F | CTGTAACATGGAAACTGGGGAAA |
| QPCR-Col3a1-R | CCATAGCTGAACTGAAAACCACC |
| QPCR-Eln-F | TTGCTGATCCTCTTGCTCAAC |
| QPCR-Eln-R | GCCCCTGGATAATAGACTCCAC |
| QPCR-Myh6-F | GCCCAGTACCTCCGAAAGTC |
| QPCR-Myh6-R | GCCTTAACATACTCCTCCTTGTC |
| QPCR-Actc1-F | CTGGATTCTGGCGATGGTGTA |
| QPCR-Actc1-R | CGGACAATTTCACGTTCAGCA |

| | |
|---|---|
| QPCR-Scn5a-F | ATGGCAAACTTCCTGTTACCTC |
| QPCR-Scn5a-R | CCACGGGCTTGTTTTTCAGC |
| cTnT F | CAGAGGAGGCCAACGTAGAAG |
| cTnT R | CTCCATCGGGGATCTTGGGT |
| QPCR-Nkx2-5-F | GACAAAGCCGAGACGGATGG |
| QPCR-Nkx2-5-R | CTGTCGCTTGCACTTGTAGC |

Supplementary Movie S1. Spontaneous contractions of iCMs from MEFs treated with MM408 at 4 weeks.

Supplementary Movie S2. Spontaneous contractions of iCMs from MEFs without treatment at 4 weeks.

Supplementary Movie S3. Spontaneous Ca2+ Oscillations in MM408-treated single iCMs from MEF labeled with Rhod-3 (Red) at 4 weeks.

Supplementary Movie S4. Spontaneous Ca2+ Oscillations in iCMs from MEFs without treatment labeled with Rhod-3 (Red) at 4 weeks.

Supplementary Movie S5. Spontaneous contractions of iCMs from neonatal cardiac fibroblast treated with MM408 at 4 weeks.

Supplementary Movie S6. Spontaneous contractions of iCMs from neonatal cardiac fibroblast without treatment at 4 weeks.

Supplementary Figure 1. Mll1 inhibitor MM408 promoted iCM formation and maturation. (A) Quantification of the number of α-actinin+ iCM loci per field by ICC. (B) ICC demonstrating the presence of cardiomyocytes cTnT+ in cells transduced with MGT after different treatments by fluorescence microscopy (400X). The right

panels are enlarged areas of the left panels. Quantification of the number of cTnT+ iCM loci per field. Error bars indicate mean ± SEM; $*p < 0.05$, $**p < 0.01$, $*** p < 0.001$.

Supplementary Figure 2. Quantification of the number of beating iCM loci with indicated viral infection and small molecule treatment for 4 weeks using neonatal cardiac fibroblast (CF) (n=5). Error bars indicate mean ± SEM; $*p < 0.05$, $**p < 0.01$, $*** p < 0.001$.

Supplementary Figure 3. Expression of adipocyte marker genes Fabp4 and Pparg during reprogramming in MGT-transduced MEFs, with or without MM408 treatment. Statistical significant difference of the expression of Fabp4 (p=0.0018) and Pparg (p=0.0288) at D11. Error bars indicate mean ± SEM; $*p < 0.05$.

Supplementary figure 1

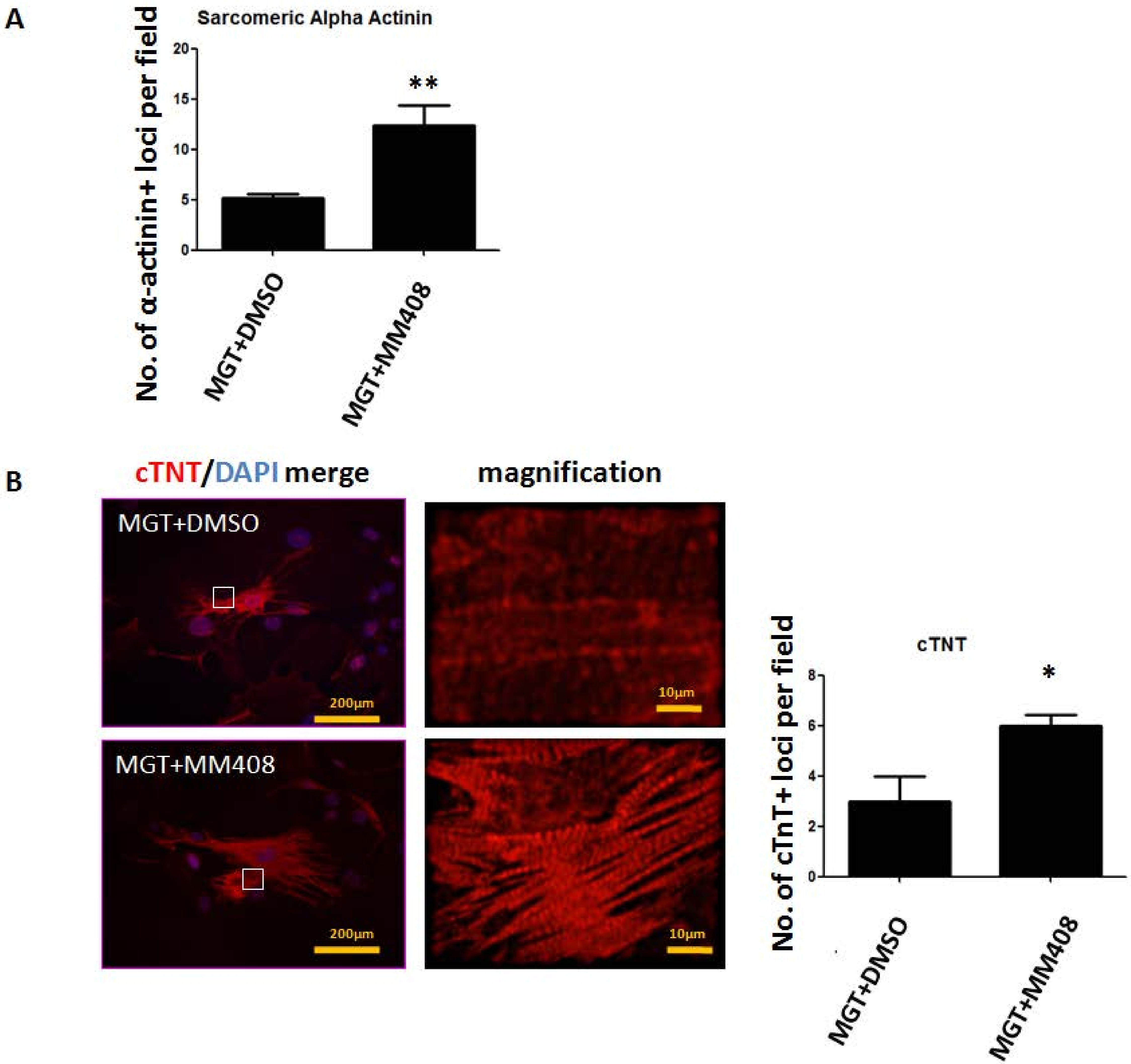

## Supplementary figure 2

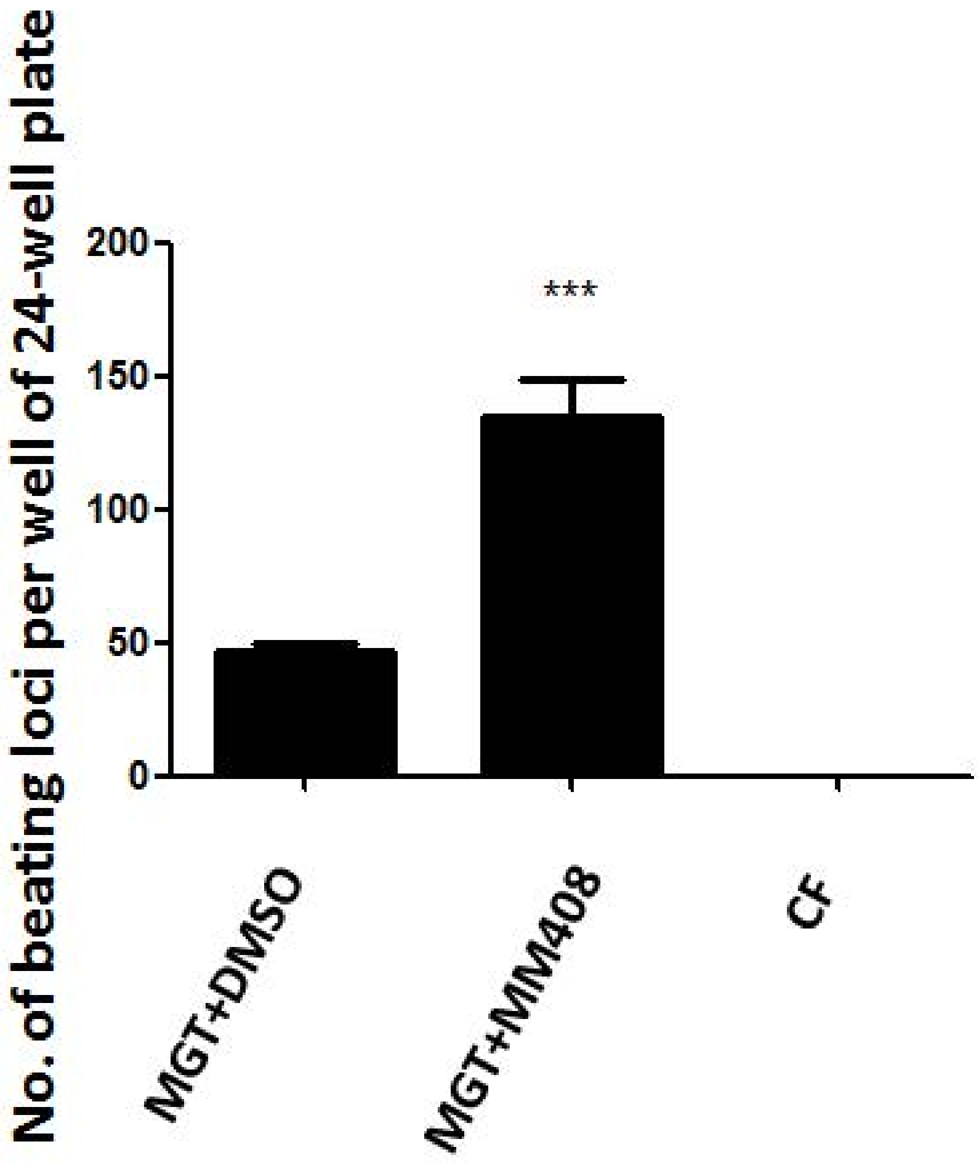

**Supplementary figure 3**

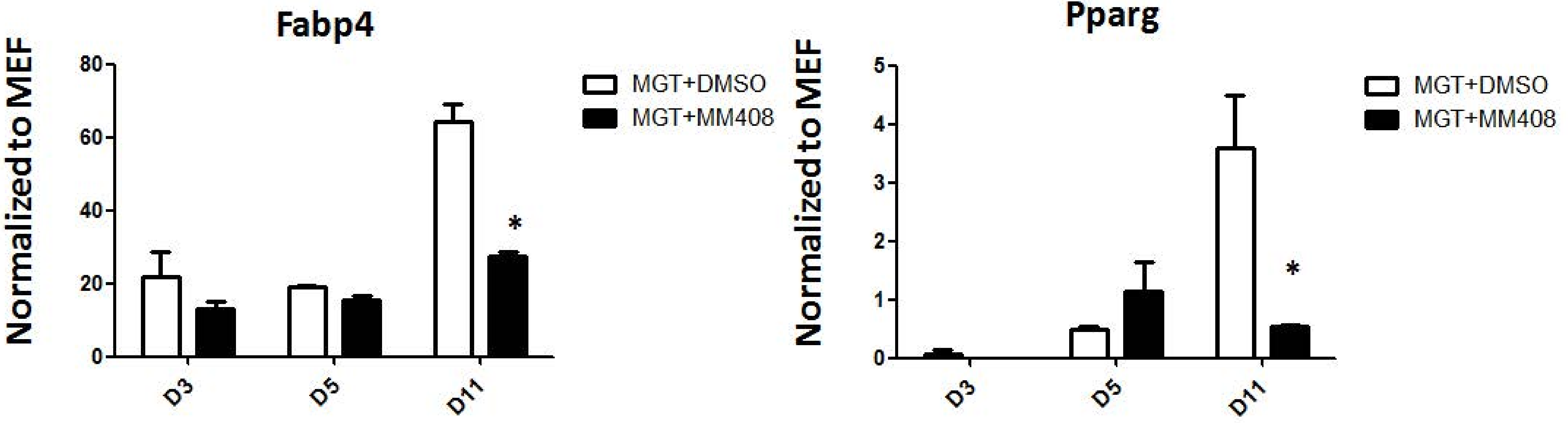

Supplement: Supplementary Information [file celldisc201636-s1.pdf]
